# Why antibody isotype matters for immunotherapy

**DOI:** 10.1093/abt/tbag032

**Published:** 2026-06-11

**Authors:** Sandra Kleinau

**Affiliations:** Department of Cell and Molecular Biology, Uppsala University, 75124 Uppsala, Sweden

**Keywords:** therapeutic antibodies, IgG/IgA subclasses, 3D-tumor model, complement-dependent cellular cytotoxicity, antibody-dependent cellular phagocytosis

## Abstract

Monoclonal antibodies (mAbs), engineered to recognize disease-associated antigens, are central to cancer immunotherapy due to their ability to block or eliminate malignant cells by engaging immune effector mechanisms. Yet many tumors evade or suppress these responses, limiting durable clinical benefit and highlighting the need to optimize antibody-based strategies. A critical determinant of mAb activity is antibody isotype, which governs interactions with Fc receptors, complement pathways, and immune effector cells. While most approved therapeutics employ immunoglobulin G1 (IgG1) for its robust immune-stimulating capacity, evidence suggests that context-dependent isotype selection could improve efficacy. Here, we review the structural and functional properties of human IgG and IgA subclasses, with a focus on anti-CD20 rituximab in two- and three-dimensional models of human B-cell lymphoma. We further discuss how antigen density, tumor architecture, and host variables influence antibody-mediated effector functions. Together, these considerations highlight opportunities to refine antibody isotype selection for more individualized cancer immunotherapy.

**Statement of Significance**

Antibody isotype is an important but often underexplored determinant of immunotherapy success. Beyond IgG1, subclasses such as IgG3, IgG2, and IgA display distinct functional properties that broaden therapeutic strategies, including agonistic combinations. Integrating insights from advanced 3D tumor models, this review highlights rational, personalized isotype selection to optimize clinical outcomes.

## Introduction

Antibody-based immunotherapy has emerged as a central approach not only in cancer but also in autoimmune and infectious diseases. Monoclonal antibodies (mAbs) have become the predominant class of biological therapeutics, targeting a wide range of antigens with high specificity and precision. Their clinical use has markedly improved survival and reshaped treatment paradigms for many malignancies. Nevertheless, a substantial proportion of patients either fail to respond or eventually relapse, highlighting the ongoing need to optimize these therapies.

The activity of an individual antibody depends on multiple factors beyond its antigen-binding affinity and epitope specificity. Features such as isotype, subclass, and glycosylation pattern critically influence how antibodies engage the immune system and mediate cellular and humoral responses. Different isotypes confer distinct functional profiles by modulating interactions with Fc receptors (FcRs) on effector cells and with the complement cascade, thereby shaping the overall immunological outcome.

Here, we focus on how antibody isotype selection can influence therapeutic efficacy, using anti-CD20 rituximab in B-cell lymphoma as a model. IgG and IgA subclasses exhibit divergent abilities to induce processes such as antibody-dependent cellular phagocytosis (ADCP), complement-dependent cytotoxicity (CDC), and direct induction of cell death. We further examine how tumour architecture, antigen density, and host immune responses modulate these effects. By integrating these insights, we highlight strategies to optimize antibody-based immunotherapy and outline potential directions for future development in this rapidly evolving field.

## Immunoglobulins

Human immunoglobulins comprise five classes that share a conserved architectural framework, built from two identical heavy (H) chains and two identical light (L) chains linked by interchain disulfide bonds (Fig. 1) [1]. The variable regions of the L and H chains together form the antigen-binding fragment (Fab), which mediates specific recognition of antigens. The heavy chain also contains multiple constant domains (CH1–CH3; CH4 in some classes), which provide structural support and contribute to effector functions. The CH2 and CH3 domains together form the crystallizable fragment (Fc), which engages FcRs and the complement system to elicit immune responses.

**Figure 1:**
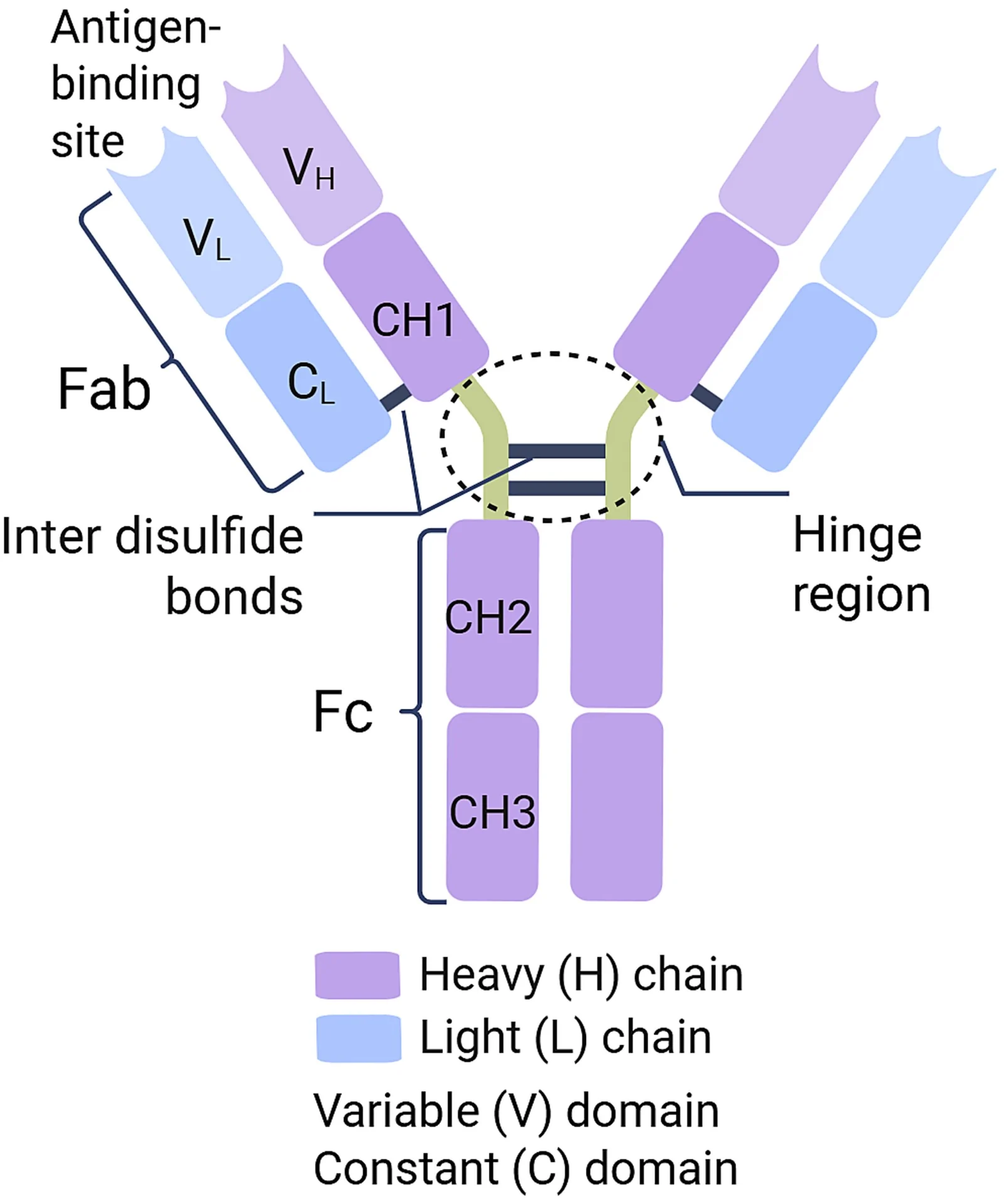
Structural organization of an antibody molecule showing heavy and light chains with variable (VH, VL) and constant (CH1–3, CL) regions. The variable regions form the antigen-binding sites within the Fab domains, whereas the Fc domain comprises constant regions that determine antibody isotype and effector functions. The hinge region provides flexibility between the Fab and Fc domains, and interdisulfide bonds stabilize the overall structure.

A defining structural element of antibodies is the hinge region, a flexible polypeptide segment connecting CH1 and CH2 that provides both structural elasticity and functional versatility (Fig. 1). This flexibility enables the Fab arms to adopt multiple orientations, allowing antibodies to recognize epitopes in diverse spatial arrangements. The hinge also affects the accessibility and positioning of the Fc region, thereby influencing how effectively the antibody engages its effector mechanisms. Residues in the CH2 domain, particularly those nearest the hinge, form a key binding site for complement components and FcRs that interact with the Fc portion of immunoglobulins [2]. These interactions underlie classical complement activation and antibody-dependent cellular responses. However, the hinge also presents a structural vulnerability: its exposed and flexible nature renders it susceptible to proteolytic cleavage, which can compromise antibody stability and function.

IgG and IgA are the most abundant antibody isotypes in humans, with IgG comprising ~80% of circulating antibodies and IgA, primarily located on mucosal surfaces and in secretions, accounting for ~15% of the total antibody pool [1, 2]. While IgG is the predominant isotype used in therapeutic antibody development, IgA has demonstrated considerable potential in preclinical studies [3], and its therapeutic application is being actively explored. Structurally, IgG contains interchain disulfide bridges within the hinge region, whereas IgA monomers possess interchain disulfide bonds that stabilize the CH2 domain (Table 1) [4]. These structural distinctions contribute to differences in flexibility, stability, and effector functions between the two antibody classes.

**Table 1:** Features of human IgG and IgA subclasses.

**Subclass characteristics**	**IgG1**	**IgG2**	**IgG3**	**IgG4**	**IgA1**	**IgA2**
**Structure**						
Hinge length (no. of amino acids)	Moderate (15)	Short (12)	Long (32–62)	Short (12)	Moderate (23)	Short (10–13)
Interchain disulfide bridges in hinge	2	4	11	2	no	no
Interchain disulfide bridges in CH2	No	No	No	No	Yes	Yes
Disulfide linkage of L to H chains	Near hinge	In fab	In fab	In fab	In fab	In fab
**Binding**						
FcγRI (CD64)	Moderate	–	High	Moderate	–	–
FcγRIIa (CD32a)	Moderate	Low^*^	Moderate	Low	–	–
FcγRIIIa (CD16a)	Moderate^*^	–	Moderate^*^	Low	–	–
FcαR (CD89)	–	–	–	–	High	High
Complement	Moderate	Low	High	–	–	Low
FcRn	High	Moderate	Low	Moderate	–	–

L, light; H, heavy; −, no or negligible; ^*^, FcγR allotype dependent.

Despite being the smallest of all immunoglobulins, IgG exhibits broad effector functions. The small size allows them to move easily across cell membranes and its versatility supports a broad range of effector functions. IgG antibodies are highly effective opsonins, neutralizing toxins and promoting phagocytosis or complement activation by binding to antigens.

In contrast, serum IgA, primarily present as monomers and functionally distinct from secretory IgA in the gut, plays specialized immunological roles [5]. Although IgA is a weaker opsonin and a less efficient activator of complement than IgG, IgA-based therapeutics have demonstrated potent phagocytic activity through engagement of FcRs on myeloid cells. Accordingly, tumor antigen–specific IgA has been shown to antagonize ovarian cancer progression, underscoring the potential of IgA-based strategies as an alternative or complement to conventional IgG approaches in targeting mucosal tumors [3].

Immunoglobulins are glycoproteins, and each CH2 domain of IgG contains an N-linked glycosylation site, which can vary between molecules and even between the two sites on the same molecule [6]. Differences in sialic acid, galactose, *N*-acetylglucosamine, and fucose influence Fc structure by modulating the spacing between CH2 domains, thereby affecting both antibody stability and function [7]. Consequently, IgG molecules lacking glycosylation due to mutations show reduced or abolished FcR binding [6].

IgA antibodies are also highly N-glycosylated, mainly on the CH2 and CH3 domains, and alterations in these carbohydrates may influence FcR clustering and signaling [5].

## Characteristics of IgG subclasses

The four human IgG subclasses, IgG1, IgG2, IgG3, and IgG4, share ~90% sequence identity, with key differences in the amino acid composition of the hinge region and upper CH2 domains (Table 1). Specifically, the hinge length varies in the following order: IgG3 > IgG1 > IgG4 > IgG2 [8]. The extended hinge of IgG3 amplifies Fab–Fab and Fab–Fc flexibility, and enhances epitope accessibility and binding valency compared to the other IgG subclasses. In contrast, IgG2 has the shortest and most rigid hinge among the IgG subclasses. IgG4 also has a relatively short hinge, with flexibility intermediate between that of IgG1 and IgG2. Furthermore, the inter-chain disulfide bridges are different in IgG subtypes [2]. Thus, in IgG1 the disulfide linkage between the C-terminal cysteine residue of the light chain connects to the first cysteine residue in the hinge of the heavy chain, whereas in IgG2, IgG3, and IgG4 subtypes, the light chain is disulfide-bonded to the cysteine residue in the Fab portion of the heavy chain (Table 1) [9].

Human IgG2 has extra interheavy chain disulfide bonds that restrict the molecule’s flexibility (Table 1). In fact, IgG2 is unique among the IgG subclasses because it can rearrange disulfide bonds in its CH1 and hinge regions, producing structurally distinct isoforms [10]. IgG2-A is the classic structure, defined by its flexible “Y” conformation, whereas the unusual disulfide hinge in IgG2-B adopts a more compact shape with the Fab′ arms held in close proximity to the hinge [11]. Notably, the IgG2-B isoform shows agonistic activity independent of FcR interactions, highlighting how subtle structural differences can significantly impact function. IgG4 can form half-molecules through stable intrachain disulfide bonds between hinge-region cysteines, and *in vivo* exchange between different IgG4 molecules can lead to a repertoire of IgG4 molecules with continuously shifting Fab domains [12].

IgG binds FcRs (FcγRs) primarily when it is complexed with antigen, and differential engagement of these transmembrane glycoproteins by individual IgG subclasses shapes both the magnitude and quality of downstream immune responses. The capacity of IgG-immune complexes to engage FcγRs is determined by the CH2 domain, together with subclass-specific features of the hinge region. As a result, the human IgG subclasses exhibit distinct affinities for the three major FcγR classes; FcγRI, FcγRII, and FcγRIII, with FcγRII and FcγRIII each comprising multiple isoforms [13].

FcγRI (CD64) is a high-affinity receptor (10^8^–10^9^ M^−1^) capable of binding both monomeric IgG1 and IgG-containing immune complexes, thereby mediating activating responses through an associated immunoreceptor tyrosine-based activation motif (ITAM) [14]. In contrast, FcγRII (CD32) and FcγRIII (CD16) are low-affinity receptors (∼10^6^ M^−1^). Among these, CD32a, CD32c, and CD16a function as activating receptors that signal via ITAM-containing adaptor proteins, whereas CD32b, which contains an immunoreceptor tyrosine-based inhibitory motif (ITIM), delivers inhibitory signals.

CD32a exists in two allotypic variants, with CD32a-H131 displaying higher affinity for IgG1, IgG2, and IgG3 than CD32a-R131 [15]. Similarly, CD16a occurs in two allotypes, of which CD16a-V158 binds IgG1 with greater affinity than CD16a-F158. Distinct from the other FcγRs, CD16b is a glycosylphosphatidylinositol (GPI)-anchored receptor and primarily mediates activation in neutrophils [14].

All FcγRs are widely expressed on leukocytes, except for lymphocytes, which express only CD32b on B cells. The overall biological response facilitated by ITAM-containing FcγRs varies depending on the cell type expressing the FcR. However, it generally involves the secretion of cytokines and inflammatory mediators, respiratory burst, ADCP, and antibody-dependent cell-mediated cytotoxicity (ADCC) [13]. ADCC is a mechanism mediated by CD16a and is largely associated with natural killer (NK) cells, which express CD16a, but no other Fcγ receptors [16].

IgG1 and IgG3 are the most potent activators of FcγRs, whereas IgG2 and IgG4 exhibit more limited effector functions (Table 1). The longer hinges in IgG1 and IgG3, compared to IgG2 and IgG4, may contribute to enhanced FcγR engagement and stronger effector functions [17]. In line with these properties, IgG2 binds poorly to most FcγRs, interacting primarily with CD32a. In contrast, IgG4 exhibits only moderate affinity for FcγRs, interacting primarily with CD64 and, to a lesser extent, with CD32a, CD32b, and CD16a.

The structural differences among IgG subclasses influence not only their effector functions but also their half-life. Thus, each subclass interacts differently with the neonatal Fc receptor (FcRn) (Table 1), which protects IgG from lysosomal degradation [18, 19]. The FcRn binding site is located at the CH2–CH3 junction, an area rich in histidines. IgG1 has the longest serum half-life (~3 weeks), followed by IgG2 and IgG4, whereas IgG3 has the shortest (~7 days) due to a unique arginine substitution that replaces histidine found in the other subclasses. After endosomal uptake of IgG3, FcRn binding is outcompeted by IgG1, leading to degradation rather than recycling. Notably, Fc engineering studies show that specific mutations can extend IgG half-life, offering strategies to reduce therapeutic dosing [20].

## Characteristic of IgA subclasses

IgA exists as two main subclasses, IgA1 and IgA2, which differ markedly in both structure and function (Table 1). IgA1 is distinguished by a longer hinge region enriched in O-linked glycans [21], conferring greater flexibility for binding antigens spaced further apart. In contrast, IgA2 lacks this extended hinge and contains fewer sialic acid residues in the CH2 domain, reflecting distinct structural and functional characteristics [5]. Comparative studies have shown that IgA2 displays a more pro-inflammatory profile, activating myeloid cells more strongly than IgA1. Notably, enzymatic removal of sialic acid from IgA1 enhances its inflammatory potential to levels comparable with IgA2, suggesting that glycosylation may play a critical role in regulating IgA effector functions.

IgA-opsonized target cells activate immune cells via cross-linking the transmembrane FcR for IgA (FcαRI; CD89). CD89 belongs to the same family as FcγRs, yet it shares only 20% sequence similarity with FcγRs. Signaling through CD89 occurs via the FcRγ adaptor chain, which contains ITAM motifs that trigger downstream signaling upon receptor crosslinking by IgA-immune complexes [22]. Both IgA1 and IgA2 subclasses bind CD89, but IgA2 engages the receptor to a higher degree than IgA1 and induce stronger effects (Table 1) [5]. CD89 is expressed by monocytes, neutrophils, eosinophils, and subsets of macrophages and dendritic cells. When CD89 is cross-linked by IgA-opsonized targets, it triggers various processes in myeloid cells, including cytotoxicity, phagocytosis, generation of reactive oxygen species, release of inflammatory mediators and chemoattractant, and the formation of neutrophil extracellular traps [23, 24].

IgA antibodies lack a binding site for FcRn and are therefore not recycled through this pathway, resulting in a relatively short serum half-life of only 4–7 days compared with IgG. Several engineering strategies have been developed to improve the pharmacokinetic properties of IgA. Increased sialylation and removal of terminal galactose residues on IgA glycans have been shown to extend serum persistence relative to wild-type IgA, underscoring the potential of glycoengineering to optimize IgA-based therapeutics [25]. Another approach involves fusion to an albumin-binding domain, enabling IgA to engage FcRn indirectly via albumin and thereby enhance systemic stability [19].

## Direct or indirect induction of cell death by antibodies

Fc-dependent immune effector mechanisms play a major role in the cytotoxic activity of therapeutic antibodies against target cells. However, certain antibodies can also induce targeted, cell-specific killing independently of immune system engagement. Through Fab-mediated antigen binding, these antibodies may trigger apoptotic or nonapoptotic cell death by crosslinking specific surface antigens, acting as agonists, or mimicking ligand-mediated activation of death receptors [26, 27]. Yet most mechanistic studies have been done in *in vitro* systems that omit critical *in vivo* variables that can alter antibody effector mechanisms.

## Complement-dependent cytotoxicity

The complement system, composed of plasma proteins and cell surface receptors, plays a key role in modulating antibody effector functions. Notably, the structural features of specific IgG and IgA subclasses, particularly the residues near the hinge region in the CH2 domain, form a significant binding site for complement components [2]. Consequently, CDC stands out as a primary mechanism by which mAbs can initiate the classical complement pathway via C1q binding to eliminate target cells. This activation results in C3b deposition for opsonization and formation of the membrane attack complex (MAC; C5–C9), culminating in cell lysis. Because the affinity of C1q for a single IgG molecule is inherently low, effective complement activation relies on avidity**,** achieved through six oligomerized IgG molecules on the cell surface. Indeed, IgG molecules that can form hexamers on target surface form an optimal platform for C1q docking and activation [28].

Among the IgG subclasses, IgG1 and IgG3 are the most potent activators of C1q and mediators of CDC (Table 1), owing to their Fc region structures (Table 1) [2]. The enhanced activity of IgG3 is attributed to its extended hinge, which allows greater flexibility in targeting antigens and engaging C1q [17]. IgG2 displays limited CDC capacity but can effectively activate complement when bound to highly repetitive or densely clustered epitopes (Table 1). On the other hand, IgG4 has minimal ability to engage complement and is generally considered noncytolytic [29]. However, this property makes IgG4 particularly advantageous for therapeutic antibody design in settings where complement-mediated inflammation should be avoided, such as in certain tumor microenvironments. IgA1 and IgA2 can bind C1q under certain conditions, but they are generally poor activators of the classical pathway. Nonetheless, IgA2 may have a greater capacity than IgA1 to induce complement-mediated target cell lysis, potentially due to the structural differences (Table 1) [30].

Furthermore, mAbs with modified glycosylation profiles can impact complement activation. Fc-galactosylation enhances C1q-binding, while fucosylation have no effect. Certain glycoforms also enable binding to mannose-binding lectin (MBL), initiating the lectin pathway through activation of MBL-associated serine proteases, mirroring the classical pathway’s downstream events [31, 32].

Complement activation is tightly regulated by membrane-bound complement regulatory proteins such as CD46, CD55, and CD59 to avoid damage to host cells [33]. Tumor cells often upregulate these complement regulatory proteins to evade CDC [34]. Consequently, the variable expression of complement regulatory proteins on tumor cells may contribute to the inconsistent clinical responses observed with therapeutic mAbs. To enhance CDC efficacy, several strategies have been explored, including engineering mAbs with optimized Fc domains to promote hexamer formation, modifying Fc glycosylation to increase complement activation, and combining mAbs with inhibitors targeting complement regulatory proteins to sensitize resistant tumor cells.

## Target cell elimination via antibody-dependent cell-mediated cytotoxicity and antibody-dependent cellular phagocytosis

Beyond complement activation, mAbs exert cytotoxic effects by engaging immune effector cells through Fc-mediated mechanisms. In these processes, antibody-opsonized tumor cells are recognized and eliminated by ADCC or ADCP, through direct interaction of the antibody’s Fc domain with the corresponding FcR. Typically, CD16a is activated during ADCC by NK cells or macrophages to eliminate IgG-opsonized target cells, either through the release of lytic granules or by inducing death receptor–mediated apoptosis [16]. ADCC can also be triggered via CD32a on myeloid-derived granulocytes. Although many anti-tumor mAbs have been shown to mediate ADCC in both two-dimensional (2D) and three-dimensional (3D) cancer models, the clinical relevance of this mechanism of action remains incompletely understood. However, surgical specimens from IgG-treated cancer patients have demonstrated significantly increased numbers of tumor-associated NK cells and elevated granzyme B expression, supporting an *in vivo* role for NK cells in the mechanism of therapeutic mAbs [35].

ADCP is the process by which antibody-opsonized target cells are internalized and degraded by phagocytes, primarily through CD32a, although other Fc receptors can also contribute to phagocytic activity [36]. The magnitude of the ADCP response depends on several factors, including the type, density, and molecular characteristics of the target antigen, as well as the structural orientation of the epitope [37]. The antibody features that determine ADCP efficiency remain incompletely defined, although the extended hinge region of IgG3 has been shown to enhance phagocytic potency [38].

Notably, ADCP represents a major effector mechanism of therapeutic IgG antibodies in oncology, with macrophages being major contributors to tumor cell clearance across preclinical and translational settings. In animal models, depletion of macrophages markedly reduces the tumor-killing efficacy of therapeutic IgG, whereas depletion of NK cells or neutrophils has minimal effect [39]. Furthermore, human macrophages, as well as neutrophils, have been shown *in vitro* to mediate efficient CD89-dependent tumor cell killing when engaged by engineered, tumor-specific IgA mAbs [40].

## Macrophages as effector cells

The effectiveness of mAbs in mediating tumor cell killing is often evaluated in cell-based assays involving human immune effector cells and tumor target cells. Human monocytes are readily isolated from peripheral blood and represent potent effector cells equipped with FcRs, chemokine receptors, and pattern recognition receptors. However, monocytes constitute a heterogeneous population, and individuals can differ in both expression levels and polymorphic variants of FcRs, as well as in other receptors, that can influence the outcome of antibody effector functions [41].

The predominant classical subset of human monocytes, identified as CD14^+^, is characterized by the expression of CD64, CD32a, and CD89. A minor subset, the nonclassical monocytes, additionally express CD16a. Classical monocytes are recruited from the circulation into tissues in response to inflammatory or homeostatic cues, where they subsequently differentiate into macrophages [42]. Upon entering peripheral tissues, monocytes may encounter pro-inflammatory cytokines such as interferon gamma (IFNγ), which can profoundly influence FcγR expression and FcγR-mediated cytotoxic antibody functions [43]. Indeed, newly immigrating monocytes can be as effective in depleting opsonized cells as tissue-resident macrophages [44].

Macrophages exhibit diverse activation states depending on the local cytokine milieu and tissue environment. However, they are often broadly categorized into two functional subsets: M1 macrophages, which promote immune activation and inflammation, and M2 macrophages, which contribute to immune regulation, wound healing, and tissue regeneration [45].

In light of macrophages positive roles in antibody-based therapeutic strategies, they are also key cells that can create an immunosuppressive tumor microenvironment. In fact, macrophages are the most abundant population of immune cells in the tumor microenvironment of most malignancies [46]. Monocyte-derived macrophages, recruited by various chemoattractants and resident macrophages from surrounding tissues accumulate inside and around tumors. These tumor-associated macrophages (TAMs) typically exhibit short half-lives and limited proliferative capacity [47]. During early tumor development, tumor-infiltrating monocytes are recruited into the tumor microenvironment and, as the disease progresses, often polarize toward the anti-inflammatory M2 phenotype. These TAMs retain a degree of functional plasticity and may acquire more pro-inflammatory characteristics upon stimulation with IgG or IgA immune complexes, particularly when acting in concert with pattern recognition receptors [48, 49]. Certain TAM subsets can thus serve as effector cells mediating cytotoxic IgG activity [50]. Importantly, the local tumor milieu dynamically regulates FcγR expression on TAMs. For instance, expression of the high-affinity FcγRI has been shown to fluctuate within the same TAM subset during tumor progression, while hypoxic tumor regions may promote upregulation of the inhibitory FcγRIIb, further shaping macrophage effector function. Nevertheless, macrophage-mediated ADCP is considered an important contributor to the clinical activity of many therapeutic antibodies. However, while activated macrophages can engulf mAb-opsonized cancer cells, their phagocytic activity can be inhibited by tumor cell expression of “self” signals such as CD47, which interacts with signal regulatory protein-α (SIRPα) on macrophages [51]. The CD47–SIRPα axis functions as a critical inhibitory checkpoint that prevents macrophage-mediated clearance of healthy cells. Disrupting this interaction by targeting either CD47 or SIRPα with blocking antibodies has emerged as a promising strategy to enhance macrophage-mediated phagocytosis of tumor cells [52].

## Therapeutic monoclonal antibodies

A broad range of therapeutic mAbs, in cancer and in autoimmune diseases, have been licensed or are in development [53]. These antibodies are commonly engineered as humanized or fully human IgG antibodies to minimize immunogenicity, since murine or chimeric antibodies often pose a higher risk of adverse anti-murine reactions in patients. IgG offers the flexibility to choose IgG subclasses for specific effector functions; however, this diversity is not fully represented in the current array of licensed antibodies, with the majority being IgG1. This is likely due to that IgG1 displays potent effector functions, has a prolonged serum half-life, and has lower production cost compared to other subclasses. Nevertheless, differences in Fc-mediated effector functions across isotypes may warrant a reassessment of this preference. IgG2 and IgG4 have been used when reduced Fc activity is preferred, though IgG4 can undergo Fab-arm exchange, creating mixed molecules that may impair therapeutic efficacy. The use of therapeutic IgG3 has been lacking likely because of reduced serum half-life and difficulties purifying IgG3 with protein A.

While IgG antibodies are the most commonly used in immunotherapy, IgA antibodies have also been developed and clinically tested, albeit primarily in specific contexts—particularly in diseases affecting mucosal sites [54]. Their unique ability to engage CD89 and mediate immune responses at mucosal surfaces makes IgA antibodies a valuable alternative or complement to IgG-based therapies, especially for conditions localized to these areas.

Although Fc-mediated mechanisms are critical for the activity of cytotoxic antibodies, the choice of variable regions can also profoundly influence their effector functions. The variable regions not only determine antigen specificity and binding affinity but can also modulate an antibody’s capacity to engage FcRs and recruit immune effector cells. Consequently, both Fc engineering and careful selection of variable regions are important considerations in optimizing the therapeutic efficacy of mAbs [55].

In general, therapeutic cancer antibodies can be categorized based on their mechanisms of action. The first group comprises antibodies that recognize tumor-expressed antigens and can mediate tumor cell killing via Fc-dependent activation of CDC, ADCC, and ADCP [56]. Although these tumor antigens may not be entirely tumor-specific, they are typically expressed on tumor cells in most, if not all, patients with a given cancer type. Tumor-targeting mAbs are often combined with chemotherapy and/or radiation therapy and are also employed in the design of bispecific antibodies that target both a tumor antigen and an additional molecule [57, 58].

A second group of mAbs function as agonists or antagonists for growth factor receptors that inhibit tumor growth by hindering receptor activation [59, 60]. These direct, immune-independent mechanisms of action expand the therapeutic utility of mAbs, particularly in tumors with limited immune cell infiltration, or resistance to Fc-mediated cytotoxicity. By combining immune-dependent and immune-independent mechanisms, therapeutic antibodies offer a multifaceted approach to tumor elimination.

The third group of antibodies represent a category that block key regulators of T-cell peripheral tolerance, including cytotoxic T-lymphocyte-associated antigen 4 (CTLA-4), programmed death-1 receptor (PD-1), and its ligand PD-L1 [61]. These antibodies function by blocking inhibitory signals that normally restrain T-cell activation. By releasing this immune suppression, they restore and enhance CD8+ cytotoxic T-cell-mediated antitumor responses, thereby enabling the immune system to recognize and eliminate cancer cells [62]. In addition to blocking inhibitory pathways, checkpoint inhibitors have been shown to exert therapeutic effects through the depletion of regulatory T cells (Tregs), which are immunosuppressive cells that limit effective antitumor immunity. In this situation, the mAbs would function much as antigen targeting mAbs discussed above, requiring activatory FcγR engagement on effector cells for deleting Tregs [63]. The use of immune checkpoint blockers has substantially improved the chances of survival in patients with metastatic cancer, such as melanoma and nonsmall lung cancer [64]. However, the benefits of immune checkpoint blockade are though limited as only a subset of patients responds to treatment, and immune checkpoint inhibitors can generate an overstimulated immune system, leading to autoimmune like adverse events [65].

## Therapeutic antibodies targeting B cells in disease

B-cell malignancies are particularly amenable to therapeutic antibodies that recognize tumor-expressed antigens. This is largely due to the presence of several surface antigens such as CD20, CD19, and CD22 that are predominantly expressed at distinct stages of B-cell development. Importantly, depletion of B cells is generally well tolerated and does not result in treatment-limiting toxicity. Among these targets, CD20 has been most extensively exploited, exemplified by rituximab, a chimeric anti-CD20 antibody comprising mouse-derived variable domains and human IgG1 constant domains. Rituximab was the first monoclonal antibody approved by the US Food and Drug Administration FDA for the treatment of B-cell malignancies [66].

CD20 is expressed on pre-B, immature, and mature B cells, but is absent on early pro-B cells and plasma cells. This transmembrane receptor is involved in B-cell activation, calcium signaling, and the regulation of proliferation and differentiation. CD20 represents a highly suitable therapeutic target, as it is not internalized, downregulated, or shed following antibody engagement [67].

Over the past two decades, rituximab has become a cornerstone treatment for diseases driven by abnormal or overactive B cells, with well-established use in non-Hodgkin’s lymphoma, particularly diffuse large B-cell and follicular subtypes, as well as in different autoimmune diseases. Rituximab can induce substantial response rates and durable remissions in certain B-cell malignancies, while in others, rituximab needs to be combined with chemotherapy (cyclophosphamide, doxorubicin, vincristine, and prednisone; CHOP) to enhance complete response, long-term remission, and cure rate [68].

Building on the success of rituximab, newer anti-CD20 mAbs have been developed to enhance therapeutic efficacy and improve tolerability. Obinutuzumab is a humanized, glycoengineered antibody that binds CD20 in a different orientation than rituximab, while ofatumumab is a fully human antibody that recognizes a unique epitope spanning both extracellular loops of CD20 [69]. All currently approved or clinically investigated anti-CD20 antibodies belong to the IgG1 isotype, owing to its capacity to activate complement and effector cells mediating ADCC and ADCP, as well as its prolonged serum half-life conferred by FcRn binding.

The clinical success of rituximab has spurred extensive research into IgG-mediated B-cell depletion. Studies in mice have shown that mononuclear phagocytes are essential for this process, as the absence of these cells markedly reduces IgG-dependent B-cell clearance [70]. Complementary *in vitro* findings demonstrate that human macrophages can phagocytose rituximab-opsonized primary lymphoma B cells [71]. Together, these findings support an important role for macrophages as effector cells in ADCP and suggest that further efforts to enhance macrophage responses to therapeutic antibodies are warranted.

Nevertheless, rituximab and newer anti-CD20 antibodies can also mediate B-cell depletion through CDC, ADCC, and direct induction of cell death. The relative contribution of these mechanisms varies among antibodies, forming the basis for their classification as type I or type II [72]. Type I antibodies, such as rituximab and ofatumumab, promote CD20 clustering within lipid rafts and potent complement activation [73], whereas type II antibodies, such as obinutuzumab, induce minimal raft formation but trigger sustained calcium influx and apoptosis [74]. Clinically, obinutuzumab has shown superior progression-free survival compared with rituximab in CD20^+^ B-cell non-Hodgkin lymphoma, albeit with higher toxicity, while ofatumumab demonstrated lower overall response rates [75].

Although rituximab is effective in B-cell malignancies, many patients fail to respond or eventually relapse, highlighting the need to better understand its antitumor mechanisms to develop more effective therapies. Most mechanistic insights into anti-CD20 antibodies come from *in vitro* studies on CD20^+^ B-cell lines and animal models, yet these do not fully capture clinical complexity.

## Preclinical three-dimensional tumor model

To determine how antibody functions can be improved to meet therapeutic needs, a thorough understanding of antibody biology is required. A major limitation to progress in immunotherapy is the availability of experimental models that recapitulate the complexity of human malignancy and immune contexture within the tumor microenvironment.

Conventional 2D culture of human cell lines, long used for *in vitro* testing of therapeutic agents, provides incomplete tissue-like processes, whereas multicellular 3D tumor spheroids (microtumors) that mimic avascular tumors are increasingly employed in pathophysiological studies and drug screening (Fig. 2). Unlike 2D cultures, they reproduce *in vivo* tumor architecture, with nutrient and oxygen gradients that create spatial heterogeneity in proliferation, quiescence, necrosis, and differentiation [76]. Indeed, such human 3D tumor models have become valuable tools in immunotherapy research, enabling assessment of therapeutic antibody efficacy in mediating CDC, ADCC, and ADCP-driven tumor cell killing (Fig. 2) [36, 77, 78]. Effector cells in tumor spheroid co-cultures can originate from human cell lines, healthy donor blood, or patient samples. A key advantage of employing a standardized effector cell line is the elimination of donor-to-donor variability, a factor that frequently introduces confounding effects and can compromise the reproducibility of functional assays. Among available models, the monocytic cell line Mono-Mac-6, which resembles classical monocytes and expresses CD64, CD32A, and CD89, provides a physiologically relevant effector system for studying IgG- and IgA-mediated immune mechanisms [79]. Mono-Mac-6 cells have been reliably used as standardized effectors in 3D tumor models treated with therapeutic antibodies [36].

**Figure 2:**
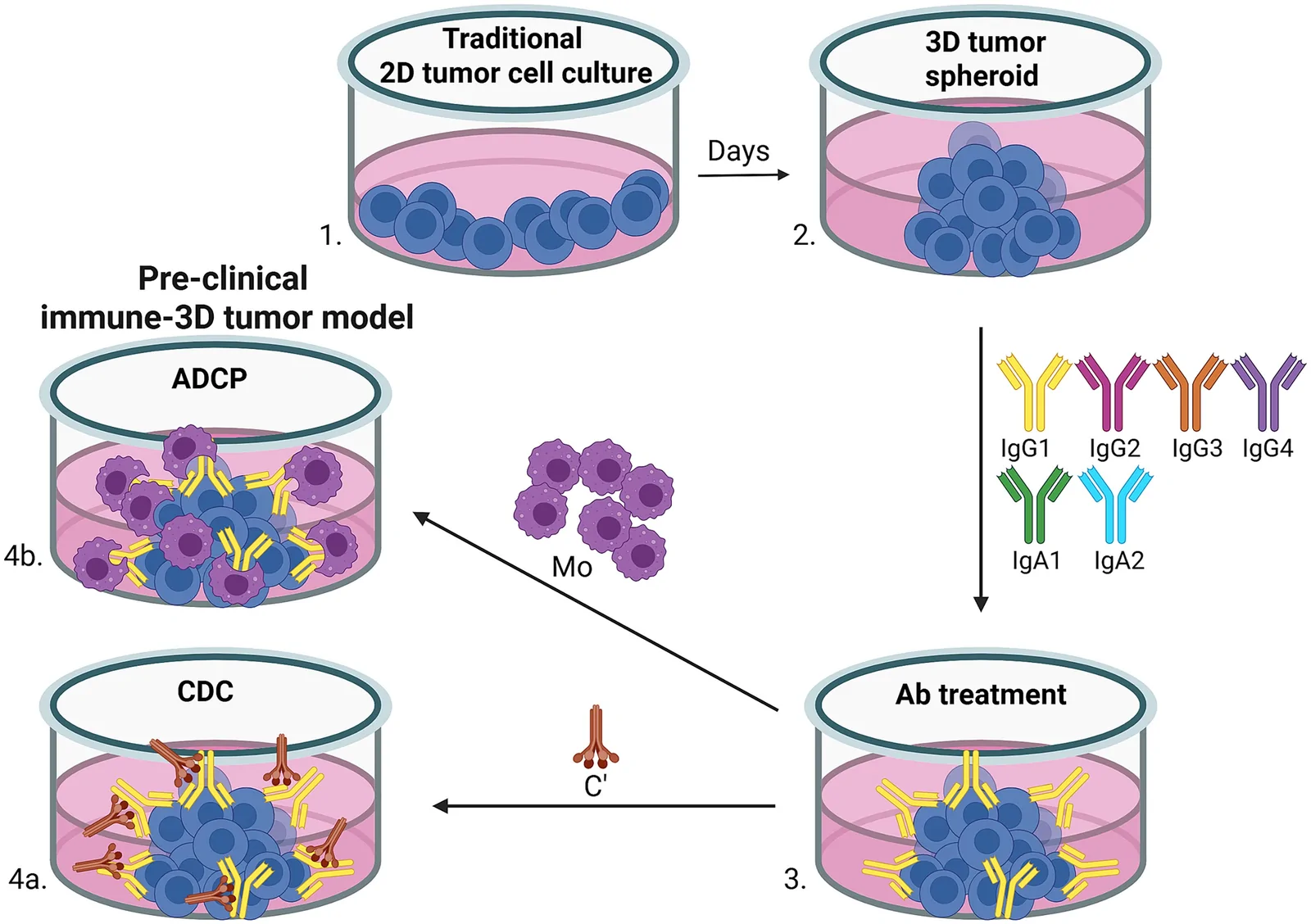
Schematic illustration of the generation of a three-dimensional (3D) immune tumor model for preclinical studies [1]. Tumor cells are cultured as a monolayer for 1–5 days to enable the formation of [2] 3D tumor spheroids [3]. Therapeutic antibodies (Abs) of different isotypes are applied separately to individual spheroids. (4a) Complement (C′)-containing medium is added to assess complement-dependent cytotoxicity (CDC). (4b) Human monocytes (Mo) are added to assess antibody-dependent cellular phagocytosis (ADCP).

A cancer spheroid immune co-culture system enables controlled investigation of antibody-dependent effector functions in a physiologically relevant context. In particular, spheroid morphology and effector–target interactions can be visualized using various imaging methods [36, 78, 80]. These approaches allow detailed assessment of tumor cells; effector cell infiltration; and spatial localization of ADCP, CDC, and other dynamic processes within the tumor microenvironment. Notably, 3D cell culture systems recapitulate the structural and functional complexity of human tissues and may, in some contexts, provide translational advantages over conventional animal models. However, 3D models remain limited in their ability to fully recapitulate the native tumor microenvironment, as they lack vascularization and fail to capture the full complexity of immune and stromal components [81]. Although co-culture approaches can partially address these limitations, immune interactions are still simplified. In addition, tumor spheroids typically exhibit reduced cellular and genetic heterogeneity compared to patient tumors, and variability in spheroid formation and culture conditions can further limit reproducibility across studies. Nevertheless, 3D models are increasingly recognized as key components of new approach methodologies, providing a valuable intermediate platform for preclinical investigation and improving the prediction of clinical responses.

## Cytotoxicity induced by rituximab isotypes

Antibody isotype and subclass have long been recognized as determinants of cytotoxic efficacy [53, 82]. However, the mechanisms of antibody activity are more complex than previously appreciated, highlighting the need to understand how structural differences influence function across subclasses and tumor microenvironments to optimize therapeutic outcomes.

While 2D cultures have long been used for preclinical B-cell lymphoma studies, recent 3D lymphoma models provide a more physiologically relevant representation of tumor architecture, cell interactions, and treatment responses [36, 83, 84], offering new insights into B-cell lymphoma therapy.

The role of CDC in rituximab-mediated B-cell depletion remains debated, as *in vitro* CDC activity does not consistently correlate with clinical response [85]. Nevertheless, studies using B-cell lymphoma monolayers demonstrate that clinically relevant concentrations of rituximab can induce CDC in the presence of complement [36, 86]. However, rituximab isotype variants differ in their capacity to induce CDC. Among human isotypes, IgG3 is the most potent, followed by IgG1, IgG2, and IgA2, whereas IgA1 and IgG4 are largely inactive in inducing CDC in 2D lymphoma cultures (Table 2) [78]. In 3D-cultured B-cell lymphoma (spheroids), rituximab IgG3 also exhibits the highest CDC activity, followed by IgG1, while other isotypes, including IgA1 and IgA2, are largely inactive. Still, compared with monolayer cultures, CDC in spheroids is largely reduced to 50% for IgG3 and 70% for IgG1, likely reflecting the structural barriers within 3D architecture. Confocal imaging of lymphoma spheroids undergoing CDC shows that apoptosis and necrosis primarily occur in the outer cell layers, indicating that complement-mediated killing is limited in the tumor core [78].

**Table 2:** Comparative *in vitro* cytotoxic potential of anti-CD20 rituximab isotypes in 2D and 3D culture of human CD20^+^ B-cell lymphoma (Raji).

**Rituximab**	**CD20+ B cell lymphoma**			
	**CDC**		**ADCP**	
	**2D**	**3D**	**2D**	**3D**
**IgG1**	+++	++	+++	++
**IgG2**	−	nd	(+)	(+)
**IgG3**	++++	+++	++++	+++
**IgG4**	−	nd	++	+
**IgA1**	−	−	++	+
**IgA2**	++	(+)	++	+

CDC, complement-dependent cytotoxicity; ADCP, antibody-dependent cellular phagocytosis—by human monocytic cells. Symbols indicate relative cytotoxic potential among isotypes within each effector mechanism**:** –, no detectable activity; (+), very low; +, low; ++, modest; +++, moderate; ++++, high; nd, not determined [36, 78, 88].

Although rituximab isotypes differ in their ability to trigger CDC, the relative pattern is preserved across B-cell lymphoma subtypes, despite variation in absolute CDC responses between individual cell lines (Table 3). These differences in CDC induction can correlate with CD20 surface density and, in particular, with the expression of complement regulatory proteins on the tumor cell [78, 87]. This regulatory relationship also appears to be influenced by the tumor microenvironment, as CD59 expression has been shown to increase under 3D culture conditions [36]. Consistent with the inhibitory role of complement regulatory proteins, depletion or blockade of CD55 or CD59 in 2D lymphoma cultures markedly enhances rituximab-mediated CDC [87]. However, in the CDC-resistant CD20^+^ B-cell line BJAB, which is characterized by high CD59 expression, specific inhibition of CD59 restores CDC activity when treated with rituximab IgG3 but not with rituximab IgG1 [78].

**Table 3:** Surface antigen expression across human CD20^+^ B-cell lymphoma cell lines and the *in vitro* cytotoxicity of anti-CD20 rituximab IgG1 in 2D culture.

**CD20+ B cell lymphoma**	**Expression**			**Rituximab IgG1**	
	**CD20**	**CD59**	**CD47**	**CDC**	**ADCP**
**Raji**	+++	+	++	+++	+++
**Granta-519**	+++	+++	++++	+	+++
**BJAB**	+	++++	++	–	+++++
**Daudi**	+	++	++	++	++++

CDC, complement-dependent cytotoxicity; ADCP, antibody-dependent cellular phagocytosis—by human monocytic cells. Symbols indicate relative expression and

cytotoxic potential within each category**:** – no activity; +, low; ++, modest;

+++, moderate; ++++, high; +++++, very high [36, 78, 88].

Collectively, these findings indicate that CDC efficacy in CD20^+^ B-cell lymphomas is governed by the interplay between rituximab isotype, CD20 antigen density, tumor architecture, and complement regulatory protein expression levels. Consequently, molecular profiling of tumor-specific features may facilitate prediction of CDC responsiveness and guide the design of optimized therapeutic strategies.

Isotype selection of therapeutic antibodies has substantial implications not only for CDC but also for ADCP. Examination of different rituximab isotypes revealed that IgG3 exhibits the strongest activity, followed by IgG1, IgG4, IgA1, and IgA2, whereas IgG2 shows minimal capacity to induce ADCP in 2D-cultured CD20^+^ lymphoma B cells (Table 2) [36]. These subclass-dependent differences are consistently observed using both Mono-Mac-6 and primary peripheral blood monocytes from healthy donors as effector populations. In Mono-Mac-6, ADCP is predominantly triggered by CD32a, as blocking this receptor markedly diminishes phagocytosis [36]. In primary monocytes, rituximab IgG1 and IgG3 induce ADCP through both CD64 and CD32a, whereas blockade of CD16a does not affect the antibody-mediated phagocytosis [41].

Notably, the ability of rituximab IgG1 to elicit ADCP in 2D-cultured lymphoma cell lines appears largely independent of the degree of CD20 expression. However, when considered alongside the inhibitory contribution of the phagocytosis checkpoint, CD47, it may influence the overall ADCP response (Table 3) [88].

The relative hierarchy of rituximab isotypes in eliciting ADCP is conserved in 3D-cultured lymphoma, though the overall phagocytic efficacy is markedly attenuated (Table 2). Among the tested variants, rituximab IgG3 shows superiority over IgG1 and additionally promotes effector cell infiltration into the spheroid core [36]. This enhanced penetration warrants further investigation of IgG3 as an alternative therapeutic format, particularly in overcoming the intrinsic treatment resistance imposed by the 3D tumor architecture [89], and underscores the need to optimize antibody engineering strategies to further improve tissue penetration and bypass microenvironmental barriers.

Overall, these findings highlight isotype and subclass as key determinants of antibody-mediated effector functions in B-cell lymphoma, with potential relevance beyond CD20 to other therapeutic antibody targets.

## Combinatorial therapies

Rituximab was initially approved as a single-agent therapy for B-cell malignancies, but it is now primarily administered in combination with cytotoxic chemotherapeutic agents, where it has markedly improved clinical outcomes. An open question, however, is whether combinatorial treatments involving different rituximab isotypes could also provide therapeutic benefit and further enhance treatment efficacy.

In monolayer cultures of CD20^+^ lymphoma cells, dual combinations of rituximab isotypes generally do not enhance ADCP beyond the effect of a single isotype but with the exception of rituximab IgG2. Notably, combining rituximab IgG2 with other rituximab IgG or IgA subclasses significantly enhances ADCP compared with each subclass alone [36, 88]. This synergistic effect is mediated by IgG2-induced, CD20-dependent apoptosis that downregulates CD47 on lymphoma cells, thereby enhancing FcγR-dependent ADCP [88]. Importantly, this mechanism enhances the tumor-killing specificity of rituximab subclasses without the off-target effects associated with anti-CD47 antibodies [90]. Anti-CD20 IgG2 can further synergize with antibodies targeting other lymphoma antigens, such as PD-L1 and CD59, to amplify ADCP [88]. These properties position rituximab IgG2 as a promising strategy to boost phagocytic activity against CD20^+^ B-cell lymphomas, leveraging apoptosis induction and CD47 downregulation in combination with FcγR-engaging antibodies. Overall, these findings highlight phagocytosis modulation as a key approach for improving cancer immunotherapy outcomes.

## Individual FcR expression and sensitivity to therapeutic antibodies

Given the variability in clinical responses to antibody-based immunotherapy, understanding how individual differences in myeloid phagocytic activity influence therapeutic outcomes has become increasingly important. Studies assessing the cytotoxic potency of different rituximab isotypes against 3D-cultured B-cell lymphomas via ADCP using peripheral blood monocytes from healthy donors have identified sex, age, and FcγR expression as key determinants [41]. Notably, monocytic FcγR expression exhibits age-dependent variation but in opposite directions between men and women [91]. Specifically, FcγRI (CD64) and FcγRIIa (CD32a) are expressed at higher levels in younger women (<54 years) than in older women (>54 years) [41], whereas older men (>54 years) display higher FcγR levels compared with younger men [41].

These findings emphasize the importance of considering biological variables in functional assays, as phagocytosis is mediated by multiple FcγRs, complicating direct comparisons of antibody-induced phagocytic efficacy. Although considerable interindividual variability in monocyte ADCP activity has been observed, this variability does not appear to depend on allelic variants of CD32a or CD16a and is only moderately influenced by FcR surface expression levels [41, 92].

Nonetheless, rituximab-mediated phagocytosis of lymphoma spheroids, particularly by IgG1, is more pronounced in monocytes from younger women (<54 years) than from older women (>54 years), while the opposite trend is observed in men. This sex- and age-associated pattern of FcR-mediated ADCP activity aligns with clinical observations: in cancer patients aged 56–66 years, male patients experienced greater therapeutic benefit from IgG antibodies than female patients [93], whereas in younger autoimmune patients (median age 46 years) treated with rituximab, sustained clinical responses were more frequently observed in women [94]. Together, these findings underscore the influence of sex- and age-related immune variation on antibody efficacy and highlight the need for more personalized approaches in antibody-based immunotherapy.

Hormone-dependent modulation of monocyte function may provide a mechanistic explanation for these treatment outcomes. Thus, *in vitro* studies have demonstrated that estrogen selectively influences monocyte activity: IgG-mediated phagocytosis is markedly reduced in monocytes from postmenopausal women but can be restored by estrogen supplementation [95]. In contrast, testosterone has been proposed to dampen macrophage-mediated phagocytosis [96]. Consequently, an age-related decline in testosterone levels may be responsible for enhanced IgG-mediated ADCP in older men. Collectively, these findings suggest that sex- and age-related immune variation may influence responsiveness to IgG-based therapies (Fig. 3a).

**Figure 3:**
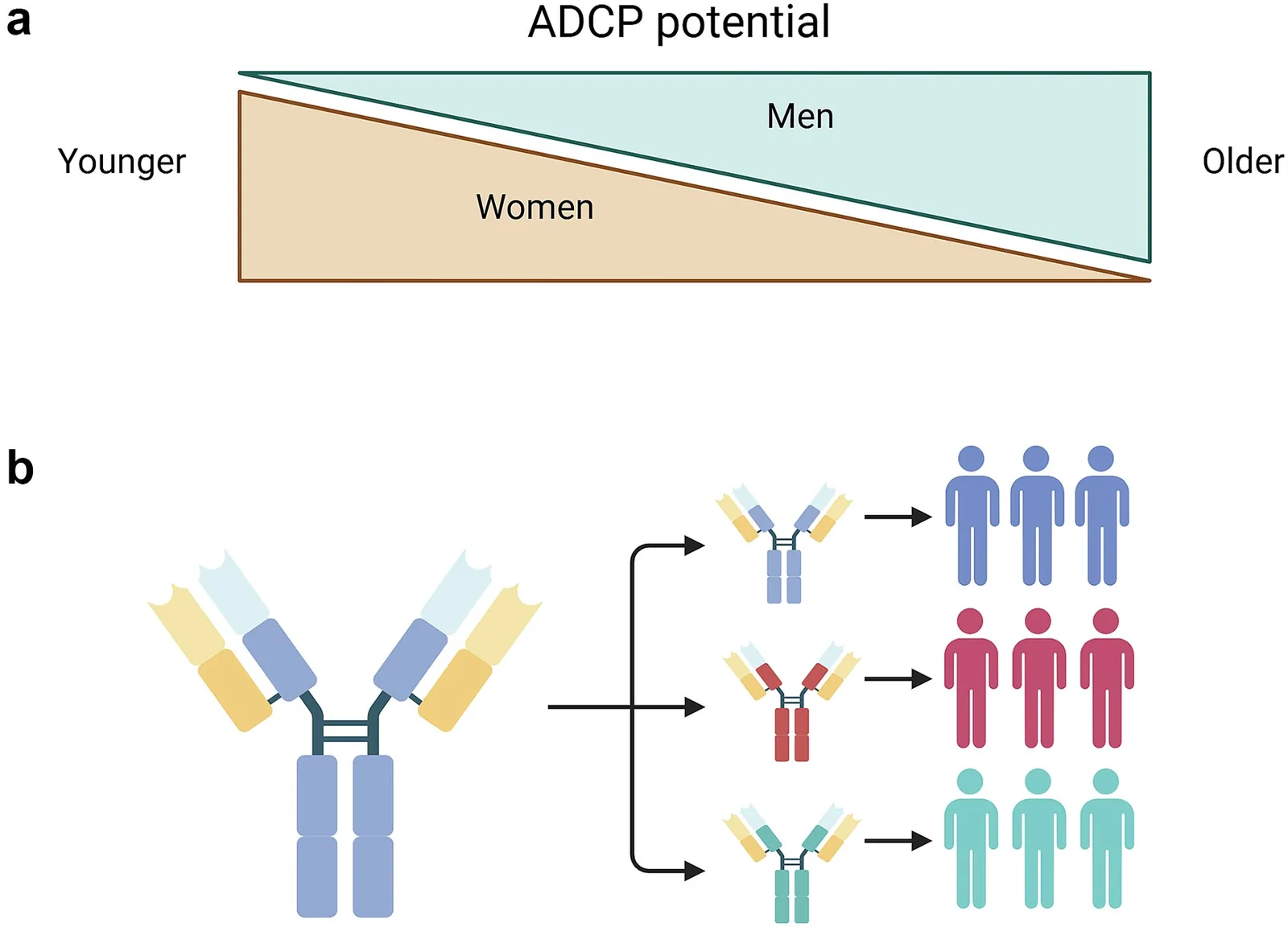
Toward individualized antibody immunotherapy. (a) Antibody-dependent cellular phagocytosis (ADCP) potency in relation to age and sex in humans [41]. (b) Schematic illustration of a therapeutic antibody engineered into different isotypes for individualized patient treatment.

## Tailoring antibody isotype for personalized treatment

Understanding individual variations in immune and myeloid phagocytic responses to therapeutic antibodies is increasingly important, given the heterogeneity of immunotherapy outcomes. A promising strategy to improve clinical efficacy may be the selective use of specific antibody isotypes in well-defined patient groups (Fig. 3). By tailoring antibody class and Fc characteristics to a patient’s sex, age, and Fc receptor profile, therapeutic outcomes may be significantly enhanced. Ultimately, the success of antibody-based therapies may depend on identifying the patients who would best respond to the treatment.

In the context of CD20-targeting therapies, improving the ability to assess rituximab effectiveness in individual patients, alongside the availability of alternative anti-CD20 antibodies, could fundamentally change how these agents are applied in clinical practice. Because many B-cell lymphoma subtypes predominantly occur in older individuals, it is important to consider potential age- and sex-related differences in ADCP. Indeed, the observation that older women exhibit reduced rituximab-mediated ADCP, whereas older men show enhanced responsiveness to rituximab-induced phagocytosis compared with younger patients, should be taken into account [41]. Recognizing and integrating these biological distinctions may inform the development of more effective, sex- and age-specific immunotherapeutic strategies. Such considerations are particularly important, as current treatment paradigms largely apply uniform approaches irrespective of patient sex or age.

A practical approach could involve selecting antibody classes tailored to specific patient groups (Fig. 3b). For example, IgG3 anti-CD20 mAbs demonstrated enhanced activity in experimental models and could therefore be of interest for further evaluation in selected patient populations, including older women. These observations raise the possibility that certain antibody isotypes may perform differently across patient subgroups. Therapeutic IgA antibodies may further expand treatment options, particularly in individuals with higher FcαRI expression, as observed in younger individuals [41].

Expanding the repertoire of antibody isotypes may refine and strengthen immunotherapy across diverse patient populations. Combining distinct isotypes and elucidating their synergistic mechanisms could further enhance cancer cytotoxicity and therapeutic efficacy. In this context, 3D models provide a robust platform for advancing personalized immunotherapy through individualized immune-based drug screening, enabling more accurate prediction of antibody activity and direct assessment of interpatient variability. Comparative evaluation of isotype potency may ultimately guide the rational design and selection of optimized, precisely tailored cancer therapies.

## Antibody engineering strategies

Antibody isotypes offer advantages in functionality and development costs. However, Fc engineering is widely used to further optimize therapeutic activity. Targeting immune cell antigens such as PD-1 and SIRPα may enhance antitumor immunity but can also cause Fc-mediated depletion of effector cells. Fc-silencing strategies (e.g. LALA mutations that reduce FcγR/C1q binding) are therefore employed to mitigate these effects, while afucosylation increases FcγRIIIa binding affinity and enhances ADCC [97, 98].

Beyond Fc optimization, protein engineering strategies such as knob-into-hole enable controlled heavy-chain pairing to generate biparatopic and bispecific antibodies. Biparatopic antibodies that bind two distinct epitopes on the same antigen can enhance affinity and functional targeting [58]. In contrast, bispecific antibodies, which engage two different antigens, typically expressed on tumor and immune cells, promote cell–cell interaction and immune activation [99]. To extend this concept, trispecific antibodies have been developed to target three epitopes, further expanding therapeutic potential in cancer immunotherapy [100]. However, increasing structural complexity may increase immunogenicity risk.

### Concluding remarks

The development of therapeutic antibodies begins with a deep understanding of their biological nature—how they function and what governs their cytotoxic potential. Each antibody is more than a mere binding molecule; its isotypic features significantly influence its ability to eliminate target cells. Accordingly, elucidating the structure–activity relationships within antibody subclasses is essential for optimizing their efficacy in immunotherapy.

Choosing isotypes with appropriate effector functions, such as human IgG3 or engineered IgG1 variants with extended hinges, may enhance therapeutic potency in relevant disease contexts. Other isotypes, including IgG2 and IgA, may also be strategically employed, with IgG2 particularly useful in combination approaches to augment effector functions through agonistic activity.

Yet, the antibody’s performance depends not only on its design but also on the tumor environment it encounters. Tumors vary, some express abundant target epitopes, others shield themselves with complement regulatory proteins or immune checkpoint molecules. Tumor architecture and microenvironment further dictate how well an antibody can reach its target and recruit immune effector cells.

The host immune system adds another layer of variability. Innate effector cells differ in their FcR profiles, influencing how they interact with antibody-coated targets. Their cytotoxic capacity can also be dampened by immunosuppressive cues within the tumor milieu, undermining ADCC and ADCP. Overcoming these suppressive forces remains a major goal for antibody-based immunotherapy.

To improve efficacy, antibody engineering has introduced numerous innovations, including humanization, as well as modifications to the protein backbone, glycans, and bispecificity. A promising future direction may be to tailor antibody treatment to individual patients, matching therapeutic format to both cancer type and immune context rather than relying on a one-size-fits-all approach.

Ultimately, therapeutic success arises from the interplay between the antibody, the target tissue, and the host immune system. Understanding and navigating this dialogue is key to designing more effective and consistent antibody therapies not only for cancer but also for autoimmune and other diseases.

## Data Availability

No new data were generated or analyzed in support of this review.
